# Doubt at the core: Unspoken vaccine hesitancy among healthcare workers

**DOI:** 10.1016/j.lanepe.2021.100289

**Published:** 2021-12-14

**Authors:** Leonardo W. Heyerdahl, Stef Dielen, ToTran Nguyen, Carla Van Riet, Tarun Kattumana, Clarissa Simas, Nico Vandaele, Anne-Mieke Vandamme, Corinne Vandermeulen, Tamara Giles-Vernick, Heidi Larson, Koen Peeters Grietens, Charlotte Gryseels

**Affiliations:** a Department of Global Health, Anthropology and Ecology of Disease Emergence Unit, Institut Pasteur, Paris, France; b Socio-Ecological Health Research Unit, Institute of Tropical Medicine, Antwerp, Belgium; c Access-To-Medicines Research Centre, KU Leuven, Belgium; d Husserl Archives, Research Center for Phenomenology and Continental Philosophy, Institute of Philosophy, KU Leuven, Belgium; e Department of Infectious Disease Epidemiology, London School of Hygiene & Tropical Medicine, London, UK; f KU Leuven, Department of Microbiology, Immunology and Transplantation, Rega Institute for Medical Research, Clinical and Epidemiological Virology, Institute for the Future, Leuven, Belgium; g Center for Global Health and Tropical Medicine, Instituto de Higiene e Medicina Tropical, Universidade Nova de Lisboa, Lisbon, Portugal; h Youth Health Care, Environment and Health Leuven University Vaccinology Centre KU Leuven, Belgium; i Vaccine Confidence Project and London School of Hygiene and Tropical Medicine, United Kingdom; j School of Tropical Medicine and Global Health, Nagasaki University, Nagasaki, Japan

Healthcare workers are a priority target population in current COVID-19 vaccination strategies because of their increased workplace exposure and contacts with potentially at-risk patients.^1^ In some European countries such as Belgium, Greece, and France,^2^ COVID-19 vaccination is now required for this group. However, studies show that a varying but often substantial proportion of healthcare workers are hesitant about receiving these vaccinations.^3^ This is extremely relevant for vaccination campaigns, as healthcare workers are among the most trusted sources of vaccine information and have a direct influence on the vaccination decisions of their patients and social contacts.^4^ Furthermore, insufficient vaccination uptake risks increasing COVID-19 infections, most likely leading to more hospitalizations and less available health staff, increasing the workload in hospitals, and thus reducing health system capacities to adequately respond to the epidemic. Health professionals often do not voice their vaccine-related concerns, particularly to colleagues, due to the institutional and societal pressures to vaccinate. We may frame this phenomenon as *unspoken vaccine hesitancy.*

This unspoken vaccine hesitancy appears at a time when people's vaccination status has become a source of widespread tension and social division within and across communities globally. The active polarization between the vaccinated and unvaccinated may further inhibit the expression of anxieties that must be addressed. Especially among healthcare workers, merely voicing vaccine-related concerns entails a risk of being lectured, mocked, stigmatized, or labeled as conspiracy theorists and ‘anti-vaxxers’. This risk is compounded by societal expectations that healthcare workers must protect individuals in their care, implying that these workers have a moral obligation to be vaccinated. This moral obligation can exacerbate pressure on health professionals who are hesitant about COVID-19 vaccines. When healthcare workers cannot express their hesitancy, their concerns become more difficult to address. In these circumstance health professionals may also face difficulties cultivating trust in Covid-19 vaccines among the lay individuals whom they attend. As such, unspoken hesitancy could reduce the core public trust in COVID-19 vaccines and vaccination programs across countries. It could also jeopardize future vaccination campaigns beyond COVID-19. Although literature is scarce on the topic, “silent refusals’ have been identified as a major challenge to vaccination uptake in Pakistan.^5^

Understanding and addressing unspoken vaccine hesitancy is one crucial but unexplored dimension of building overall vaccine confidence in and through healthcare institutions and services. Novel, pro-active, and transdisciplinary approaches are required to identify the most effective responses to this urgent challenge. Giving voice to vaccine concerns in a constructive dialogue will contribute to individual and societal well-being and resilience.

## Declaration of interests

CG, KP, SD, TK, TN, CVR, LWH report a grant from Fonds Wetenschappelijk Onderzoek (FWO- Research Foundation – Flanders), to conduct social listening of vaccine concerns in Belgium.

CS reports a grant from Johnson & Johnson and a grant from GSK on Research on Vaccine hesitance in different European countries.

HL reports a grant from Merck on Research on Vaccine hesitancy among health care providers; from GSK on Research on Vaccine acceptance during pregnancy and honoraria for a training session; a grant from Astra Zeneca to run webinars with health care professionals on covid vaccination.

CV; NV; TGV; AV have nothing to disclose.
